# 

**Published:** 1891-07

**Authors:** 


### LITERARY.

PASSION FLOWERS AND THE CROSS.—By Emma Howard Wright. Published by the Calendar Pub. Co., Baltimore. 247 pp., price 50 cents.

This is a tale of marriage without love and of love without marriage. The former a union induced on the part of the wife by the lure of wealth and social position, brought only a formal recognition of wifely attributes—a home only in name and an offspring that never knew a touch of maternal tenderness. The one bright and shining picture is the devotion of a college chum to his friend, whose premature death left a not unwilling widow an ample fortune, an only child, and a home which no embellishments of art could compensate for parental indifference, culminating in an unholy passion that penetrated the sacerdotal robe and lured a forsworn priest to his destruction. The authoress, whose likeness ornaments a fly-leaf, is evidently a woman who has keenly observed, if she has not felt, the play of passion in its various moods, and she has drawn a picture in many respects true to the life; making New York city contribute to her plot in a way not altogether strange to her Tammany-ridden precincts.

PROTECTION OR FREE TRADE.—By Henry George. Published by Henry George & Co., 42 University Place. Single copies 25 cents, 10 copies for $1.

Mr. George is the author of several works on Political Economy, which have given him a widespread reputation. The present work is an able and elaborate exposition of the Tariff, from the standpoint of absolute free trade, and has already taken its place as a political text-book of the free-traders.

THE DIOS CHEMICAL CO., OF ST. LOUIS, have issued a handsome colored chart of the UTERUS and its APPENDAGES, which they offer to furnish free to members of the medical profession on application. They will be likely to have no lack of patronage on these terms.

LYDIA E. PINKHAM A VERITABLE PERSONALITY.—The Boston Herald, of June 1st, has a column article devoted to proofs of the personal identity of the late Lydia E. Pinkham, with the well known compound so extensively advertised and sold under her name and likeness. The history of the Pinkham family is fully gone into by a son, and an original likeness of the mother shown to the Herald reporter, which he at once recognized as the same in features as the one so extensively circulated in advertising one of the most valuable remedies of the household.

TWENTY-FIVE YEARS OF BREWING.—The name of George Ehret is oftener drank in excellent beer than that of any other in New York city; and now, after having so long administered consolation to his thirsty patrons, he has attacked the brain with the most interesting history of American Beer we have seen. The work is a quarto of 120 pp., elegantly and profusely illustrated, following the Brewing industry from the barley-field to Hell-(gate). The statistics given in this pains-taken work will be interesting to hop growers, brewers, consumers, and apostles of total abstinence. The writer has fairly pictured a two-sided subject, and given it an unwonted interest.

FOR NERVOUSNESS USE HORSFORD'S ACID PHOSPHATE.—Dr. W. C. Hanscome, Minneapolis, Minn., says: "I used it in a case of acute rheumatism, during convalescence; the particuar symptoms I wished to relieve were sleeplessness and nervousness, and the results were all I desired."

ANGELS' FOOD.—By Basil Fritcher. This is an argument for a vegetarian food. to the entire exclusion of the flesh of animals, brief and to the point.

THE MICROSCOPE. An essay by Dr. H. M. Whelpley, F. R. M. S., giving much useful information.

NEW METHODS IN SURGERY.—Illustrated by A. V. L Brokaw, M. D., St. Louis. Also, the use of Segmented Rubber Rings in operating, etc., etc.

THE DUTY THE PUBLIC OWES THE PHARMACIST.—A Valedictory Address. By Prof. H. M. Whelpley, M. D., P. H. G., St. Louis.

CLIMATE OF WASHINGTON, issued by the STATE BOARD OF TRADE.

NOTE.—We received a number of additional works, including the announcement of the MEDICAL CONVENTION to be held this month, with a list of themes to be discussed, which were left upon our round-table, to be properly noticed, but filched and carried away by some meddlesome interloper during our temporary absence, thus defeating our purpose.

---

### "SHUT YOUR MOUTH."

For Hall's Journal of Health.

George Catlin wrote a pamphlet entitled "Shut your Mouth," to show the bad effects of breathing through the open mouth. He did not tell us how to keep the mouth shut during sleep.

I have seen an apparatus imported from abroad, at a cost of 75 cents, patented, like the headstall of a bridle, to put on at night.

I take a clean white cotton string, 15 or 20 inches long, put the middle part of it in my mouth and tie it loosely on my neck, just behind my mouth, my lips and tongue seize it and hold it, as long as it is in my mouth. This process is not patented and costs nothing. H. F. P.

Circleville, Ohio.

